# Breastmilk microbiome changes associated with lactational mastitis and treatment with dandelion extract

**DOI:** 10.3389/fmicb.2023.1247868

**Published:** 2023-11-13

**Authors:** Xinyan Jin, Jinhe Xiao, Chunli Lu, Wenxin Ma, Yingyi Fan, Xue Xue, Yaru Xia, Nana Chen, Jianping Liu, Xiaohua Pei

**Affiliations:** ^1^Centre for Evidence-based Chinese Medicine, Beijing University of Chinese Medicine, Beijing, China; ^2^Beijing University of Chinese Medicine Affiliated Xiamen Hospital, Xiamen, China; ^3^Department of Prevention and Treatment of Breast Disease, Haidian District Maternal and Child Health Care Hospital, Beijing, China; ^4^Guangdong Provincial Research Center of Integration of Traditional Chinese Medicine and Western Medicine in Metabolic Diseases (Institute of Chinese Medicine), Guangdong Pharmaceutical University, Guangzhou, China; ^5^Department of Breast Surgery, Beijing University of Chinese Medicine Third Affiliated Hospital, Beijing, China; ^6^The First Clinical Medical School, Hubei University of Chinese Medicine, Wuhan, China

**Keywords:** 16S rRNA gene sequencing, antibiotic, mastitis, lactation, breastmilk, Pugongying granule, Chinese herbs

## Abstract

**Introduction:**

Dandelion (Pugongying) is one of the most frequently used Chinese herbs for treating lactational mastitis (LM). Pugongying granules, a patented medication primarily comprised of dandelion extract, have been approved by CFDA for LM treatment in China. The aims of this study were to investigate the etiology of LM and the mechanism by which Pugongying granules decrease LM symptoms, with a particular focus on the microbial communities found in breastmilk.

**Methods:**

Participants were recruited from a previously performed randomized controlled trial (Identifier: NCT03756324, ClinicalTrials.gov). Between 2019 and 2020, women diagnosed with unilateral LM at the Beijing University of Chinese Medicine Third Affiliated Hospital were enrolled. In total, 42 paired breastmilk samples from the healthy and affected breasts of the participants were collected. Additionally, 37 paired pre- and post-treatment breastmilk samples from the affected breast were collected from women who received a 3-day course of either Pugongying granules (20 women) or cefdinir (17 women). Clinical outcomes [e.g., body temperature, visual analogue scale (VAS) score for breast pain, the percentage of neutrophils (NE%)] were analyzed pre- and post-treatment, and the breastmilk samples were subjected to 16S rRNA gene sequencing to analyze the alpha and beta diversities and identify significant bacteria. Finally, the relationship between microorganisms and clinical outcomes was analyzed.

**Results:**

There was no significant difference in fever and pain between the Pugongying group and cefdinir group. The most prevalent bacterial genera in breastmilk were *Streptococcus* and *Staphylococcus*. Compared to healthy breastmilk, microbial diversity was reduced in affected breastmilk, and there was a higher relative abundance of *Streptococcus*. After Pugongying treatment, there was an increase in microbial diversity with significantly higher abundance of *Corynebacterium*. A negative correlation was found between *Corynebacterium*, VAS score, and NE%. Treatment with cefdinir did not affect microbial diversity. Taken together, our results show a correlation between LM and reduced microbial diversity, as well as an increased abundance of *Streptococcus* in affected breastmilk.

**Conclusion:**

Pugongying granules enhanced microbial diversity in breastmilk samples. Given the substantial variation in individual microbiomes, identifying specific species of *Streptococcus* and *Corynebacterium* associated with LM may provide additional insight into LM pathogenesis and treatment.

## 1. Introduction

Lactational mastitis (LM) is a common condition among lactating women that may lead to breastfeeding difficulties, impacting both the mother and infant, while also increasing healthcare costs (World Health Organization, 2000; Cooney and Petty-Saphon, 2019; Fernandez et al., 2020). LM typically manifests as localized erythema, pain, and swelling of one or both breasts, and is sometimes accompanied by symptoms of general malaise, such as fever and fatigue (World Health Organization, 2000). The prevalence rate of LM among breastfeeding mothers is approximately 18 to 25% worldwide, and approximately 20 to 35% of breastfeeding mothers suffer from it more than once (Jonsson and Pulkkinen, 1994; Kinlay et al., 1998; Vogel et al., 1999; Foxman et al., 2002; Scott et al., 2008). The actual prevalence of LM may be even higher due to study design limitations, particularly with regards to observational studies.

The etiology of LM, which has not yet been fully explored, is thought to involve one or more of the following factors: breastmilk stasis, microbial infection, and microbial dysbiosis. According to the World Health Organization, breastmilk stasis and infection are the primary causes of mastitis (World Health Organization, 2000). However, breastmilk is known to harbor diverse microbial communities with recognized significance for human health. Mastitis pathogenesis has been shown to be associated to a diminished inhibitory effect of symbiotic bacteria on pathogens, including *Staphylococcus aureus* or *Corynebacterium*, an imbalance in beneficial microorganisms with depletion of anaerobic bacteria, and an increase in the abundance of conditional pathogenic bacteria coupled with a reduction in microbial diversity (Delgado et al., 2008; Jiménez et al., 2015; Sam Ma et al., 2015; Mediano et al., 2017; Patel et al., 2017).

Antibiotics are first-line treatment for LM, and their application in clinical practice varies greatly due to different national and regional policies (World Health Organization, 2000; Amir and Academy of Breastfeeding Medicine Protocol Committee, 2014). However, antibiotic regimens may not be the most appropriate treatment in some cases, as the indications for their utilization, as well as their benefits, are unclear. Because human breastmilk is non-sterile, conventional techniques that rely on culturing and counting leukocytes to diagnose LM are inadequate (Ng et al., 2004; Kvist et al., 2008). In addition, a Cochrane systematic review showed that there was insufficient evidence to conclude that antibiotics are effective for treating mastitis in breastfeeding women (Jahanfar et al., 2013). Furthermore, there does not appear to be a correlation between bacterial count and the occurrence or symptoms of LM (Kvist et al., 2008). Additionally, using antibiotics to treat LM could lead to an increase in drug-resistant strains in breastmilk, and the spread of drug-resistant bacteria from mothers to infants could lead to both excessive financial costs and unforeseeable health risks (Jahanfar et al., 2013; Marin et al., 2017; Prescott et al., 2021).

Complementary medicine, especially traditional Chinese medicine, is a potentially effective approach for LM treatment (Afshariani et al., 2014; Zhang et al., 2018). Dandelion (Pugongying) is the most commonly used Chinese herb for treating LM (Chinese Pharmacopoeia Commission, 2020; Jin et al., 2021). The main ingredient of Pugongying granules is dandelion extract, and the indications listed on the Pugongying granule package insert include LM treatment (Chinese Pharmacopoeia Commission, 2020). Furthermore, the Chinese Food and Drug Administration (CFDA) approved the use of Pugongying granules for treating LM in 2015. Pharmacological investigations have revealed that dandelion extract can inhibit the TLR2-NF-κb/MAPKs pathway, thereby protecting mammary tissue against *S. aureus* infection (Chen et al., 2021; Ge et al., 2021), suggesting that it has antimicrobial properties. In addition, dandelion has been shown to regulate lincomycin-induced dysbiosis of the intestinal microbiome in mice (Zhou et al., 2022), suggesting that it supports the native flora. However, the mechanism by which dandelion (Pugongying) improves the symptoms of LM, and particularly whether it alters local microbial communities remains unclear.

In this study, we collected paired breastmilk samples from both the healthy and affected breasts of women with unilateral LM. Additionally, breastmilk samples from the affected breast pre- and post-intervention with Pugongying or cefdinir were analyzed. We performed 16S rRNA gene sequencing with two objectives: (1) to investigate the etiology of LM by analyzing breastmilk microbial communities, and (2) to analyze the impact of Pugongying on breastmilk microbial communities and explore potential mechanisms, utilizing cefdinir as a control.

## 2. Materials and methods

### 2.1. Study design

Participants were recruited from the Beijing University of Chinese Medicine Third Affiliated Hospital (Beijing, China) between 2019 and 2020. The study was approved by Ethics Committee of Beijing University of Chinese Medicine Third Affiliated Hospital (Approval No. BZYSY-SFKTPJ-1). Written informed consent was obtained from all participants. Participants who received Pugongying and cefdinir were recruited from a previously performed three-arm randomized controlled trial (Identifier: NCT03756324, ClinicalTrials.gov).

### 2.2. Study population

#### 2.2.1. Inclusion criteria

(1) Women who were diagnosed with unilateral LM and wanted to continue breastfeeding; (2) development of LM within the past 72 h, with no abscess detected on B-ultrasound examination; (3) moderate, localized pain (with visual analogue scale (VAS) score of ≥4 points) and body temperature between 37.2°C and 40°C; (4) no other medications taken during the episode; (5) voluntarily participation and a signed informed consent form.

#### 2.2.2. Exclusion criteria

(1) Administration of various drugs and supplements, including antibiotics, probiotics, prebiotics, laxatives, antispasmodics, and antidiarrheals (e.g., lactulose and orlistat) during the 4-week period preceding enrollment and throughout the study; (2) unhealthy habits, such as smoking and drinking; (3) complications such as amebic dysentery, chronic intestinal inflammatory disease, diabetes, hyperlipidemia, renal dysfunction, liver dysfunction, immune deficiency disease, gallbladder or pancreas disease, etc.

### 2.3. Interventions and clinical outcomes

Participants allocated to the Pugongying group took 15 g of Pugongying granules three times a day for 3 days. Participants allocated to cefdinir group took a 0.1 g cefdinir capsule three times a day for 3 days. The Pugongying granules were produced by Kunming Pharmaceutical Factory Co. Ltd. (Kunming, China; CFDA Approval No. Z53020871). The cefdinir capsules were produced by Astellas Pharma Inc. (Tokyo, Japan).

The patients’ demographic and other characteristics were recorded at baseline. Clinical outcomes were recorded at baseline and after 3 days of treatment, including body temperature, VAS scores for breast pain, white blood cell (WBC) count, percentage of neutrophils (NE%), percentage of lymphocytes (LYM%), and C-reactive protein (CRP) level.

### 2.4. Collection of breastmilk samples

Paired breastmilk samples were collected from the healthy and affected breasts of women diagnosed with unilateral LM, as well as from the affected breast of pre- and post-intervention with Pugongying or cefdinir. Briefly, 10–15 ml breastmilk was manually collected into an aseptic centrifugal tube by researchers with sterile gloves, and the first few drops of breastmilk were discarded to avoid bacterial contamination from the skin. The samples were centrifuged at a low temperature at 1,300 rpm for 10 min. The supernatants were then discarded, and the remaining samples were stored at −80°C. Centrifugation and storage were completed within 2 h of collecting the samples. Subsequently, the breastmilk samples were transferred to Majorbio Bio-Pharm Technology Co., Ltd. (Shanghai, China) in liquid nitrogen for 16S rRNA gene sequencing.

### 2.5. 16S rRNA sequencing (Miseq)

#### 2.5.1. DNA extraction

Total microbial genomic DNA was extracted from the breastmilk samples using a FastDNA® Spin kit for Soil (MP Biomedicals, CA, USA) according to the manufacturer’s instructions. The quality and concentration of the DNA were determined by 1.0% agarose gel electrophoresis and a NanoDrop® ND-2000 spectrophotometer (Thermo Scientific Inc., MA, USA), respectively, and the samples were stored at −80°C for later use.

#### 2.5.2. Polymerase chain reaction (PCR) amplification

The hypervariable V3–V4 regions of the bacterial 16S rRNA gene were amplified with the following primer pair using ABI GeneAmp®9700 PCR thermocycler (ABI, CA, USA): 338F (5′-ACTCCTACGGAGGCAGCAG-3′) and 806R (5′- GACTACHVGGGTWTCTAAT-3′). The PCR conditions were as follows: initial denaturation at 95°C for 3 min; 27 cycles of denaturation at 95°C for 30 s, annealing at 55°C for 30 s, and extension at 72°C for 30 s; followed by final extension at 72°C for 10 min; and finally storage at 4°C. The components added to the PCR reaction mixture were as follows: TransStart FastPfu Buffer (5×) in a volume of 4 μl, dNTPs (2.5 mM) in a volume of 2 μl, forward and reverse primers (5 μM) in a volume of 0.8 μl, TransStart FastPfu DNA Polymerase in a volume of 0.4 μl, and template DNA (with a final concentration of 10 ng) to reach a total volume of 20 μl. All PCR reactions were performed in triplicate. The PCR products were purified using AMPure®PB beads (Pacific Biosciences, CA, USA) and quantified using a Quantus™ Fluorometer (Promega, WI, USA).

#### 2.5.3. Illumina Miseq sequencing

Purified amplicons were pooled in equimolar amounts and paired-end sequenced on an Illumina MiSeq PE300 platform (Illumina, San Diego, USA) according to the standard protocols of Majorbio Bio-Pharm Technology Co. Ltd. (Shanghai, China). The raw sequencing reads were deposited in the NCBI Sequence Read Archive (SRA) database (Accession Number: PRJNA999935).

#### 2.5.4. Data processing

Raw Fastq files were de-multiplexed using an in-house perl script, then quality-filtered by Fastp version 0.19.6 and merged by FLASH version 1.2.11 with the following criteria: (i) the 300-bp reads were truncated at any site receiving an average quality score of <20 over a 50-bp sliding window, truncated reads shorter than 50 bp were discarded, and reads containing ambiguous nucleotide were also discarded; (ii) only sequences that overlapped by at least 10 bp were assembled. The maximum mismatch ratio of the overlap region was 0.2. Reads that could not be assembled were discarded; (iii) samples were identified by the barcode and primers, the sequence direction was adjusted, exact barcode matching was performed, and two-nucleotide mismatches were allowed for primer matching. Then, the optimized sequences were clustered into operational taxonomic units (OTUs) using UPARSE 11 with 97% sequence similarity level. The most abundant sequence for each OTU was selected as a representative sequence. To minimize the effects of sequencing depth on alpha and beta diversity measures, the number of 16S rRNA gene sequences from each sample was rarefied based on minimum sequence, which still yielded an average Good’s coverage of 99%. The taxonomy of the representative sequence for each OTU was analyzed against the 16S rRNA gene database (Silva v138) using RDP Classifier version 2.13 and a confidence threshold of 0.7.

### 2.6. 16S rRNA sequencing (PacBio)

To investigate the microbial communities at the species level, full-length 16S rRNA gene sequencing of breastmilk samples was performed.

#### 2.6.1. DNA extraction and DNA amplification

Data extraction was performed as for Miseq sequencing. The bacterial 16S rRNA genes were amplified using the universal bacterial primers 27F (5′-AGRGTTYGATYMTGGCTCAG-3′) and 1492R (5′-RGYTACCTTGTTACGACTT-3′). Primers were tailed with PacBio barcode sequences to distinguish each sample. Amplification reactions (20-μl volume) consisted of 5× FastPfu buffer 4 μl, 2.5 mM dNTPs 2 μl, forward primer (5 μM) 0.8 μl, reverse primer (5 μM) 0.8 μl, FastPfu DNA Polymerase 0.4 μl, template DNA 10 ng, and DNase-free water. The PCR conditions were as follows: initial denaturation at 95°C for 3 min, followed by 27 cycles of denaturation at 95°C for 30 s, annealing at 60°C for 30 s, and extension at 72°C for 45 s, then a final extension step at 72°C for 10 min, and storage at 4°C (ABI GeneAmp®9700 PCR thermocyclerm, CA, USA). PCR reactions were performed in triplicate. After electrophoresis, the PCR products were purified using the AMPure® PB beads (Pacifc Biosciences, CA, USA) and quantified using a Quantus™ Fluorometer (Promega, WI, USA).

#### 2.6.2. PacBio sequencing

Purified products were pooled in equimolar amounts, and a DNA library was constructed using the SMRTbell prep kit 3.0 (Pacifc Biosciences, CA, USA) according to PacBio’s instructions. The purified SMRTbell libraries were sequenced on the Pacbio Sequel IIe System (Pacifc Biosciences, CA, USA) by Majorbio Bio-Pharm Technology Co. Ltd. (Shanghai, China). The raw sequencing reads were deposited in the NCBI Sequence Read Archive (SRA) database (Accession Number: PRJNA1027787).

#### 2.6.3. Data processing

PacBio raw reads were processed using the SMRTLink analysis software (version 11.0) to obtain high-quality Hifi reads with a minimum of three full passes and 99% sequence accuracy. Hifi reads were barcode-identified and length-filtered. Sequences with a length <1,000 or >1,800 bp were removed. The Hifi reads were clustered into OTUs using UPARSE 11 with 97% sequence similarity level. The most abundant sequence for each OTU was selected as a representative sequence. The taxonomy of the representative sequence of each OTU was determined by comparing with the 16S rRNA gene database (Silva v138) using RDP Classifier version 2.13 against and a confidence threshold of 0.7.

### 2.7. Bioinformatics analysis

The bioinformatics analysis was carried out using the Majorbio cloud platform.^1^ Three comparisons were made: paired breastmilk samples from both the affected and healthy breast of cases with unilateral LM; paired breastmilk samples from the affected breast pre- and post-treatment with Pugongying; and paired breastmilk samples from the affected breast pre- and post-treatment with cefdinir. The rarefaction curve, along with alpha diversity indices such as observed species (Sobs), ACE, Shannon, Simpson, Chao, and Good’s coverage, were analyzed using Mothur 1.30.1 software. Differences in alpha diversity were identified using Wilcoxon signed rank-sum test with Bonferroni correction applied to all comparisons. To visualize microbial communities among different comparisons, partial least squares discriminant analysis (PLS-DA) was carried out using the misOmics package. To investigate differences in microbial communities between comparisons, analysis of similarities (ANOSIM) was conducted based on weighted normalized unifrac distance. The Wilcoxon signed rank-sum test was used to explore significant bacterial genera among the three comparisons. Since there was a multicollinearity problem among the clinical parameters, the variance inflation factor (VIF) analysis was estimated with the vif function in the Vegan v2.4.3 package.^2^ The analysis was performed to identify significantly different clinical outcomes between pre- and post-treatment samples using distance-based redundancy analysis (db-RDA) via the Vegan 2.4.3 package. The clinical outcomes with substantial differences were then explored for their relationship with microorganisms by Multivariate Association with Linear Models (MaAsLin) analysis. Linear regression analysis was applied to determine the association between major clinical parameters identified by db-RDA analysis and alpha diversity indices (Shannon and Simpson).

### 2.8. Statistical analysis

SPSS 28.0 software (IBM, Armonk, NY, USA) was used to analyze the participants’ demographic and other characteristics, as well as clinical outcomes. Continuous variables were described using mean and standard deviation (SD), or median (M) and inter quartile range (IQR). For the Pugongying group and the cefdinir group, t-test or Mann–Whitney-U test was utilized. Additionally, for both groups, paired t-test or Wilcoxon signed rank-sum test was employed to evaluate pre- and post-treatment differences. Dichotomous variables are described as Yes or No. Chi-square test or Fisher’s exact test was utilized to analyze differences between the Pugongying group and the cefdinir group after a 3-day treatment period. *p* < 0.05 was considered statistically significant.

## 3. Results

### 3.1. Participants’ characteristics and clinical outcomes

**Figure 1:**
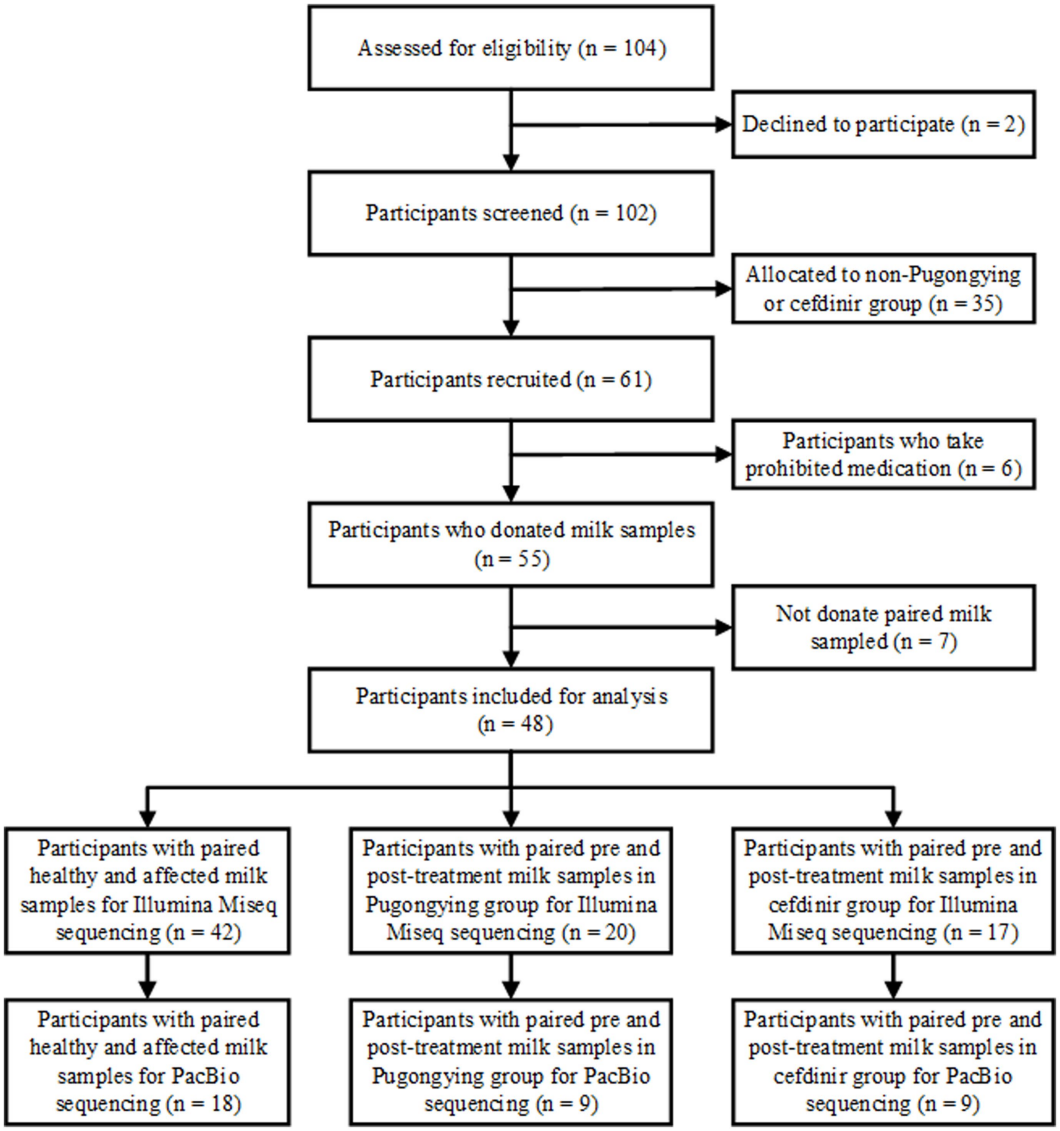
Flowchart of the study design.

A total of 127 breastmilk samples were collected from 48 lactating women who had been diagnosed with unilateral LM. A flowchart of the study design is shown in Figure 1.

A total of 84 breastmilk samples (paired affected and healthy samples) were collected from 42 participants (Table 1). Additionally, 74 pre- and post-treatment breastmilk samples were collected from 37 participants, including 20 women in the Pugongying group and 17 in the cefdinir group. Table 1 details the characteristics and demographic variables of the participants, indicating that those in the Pugongying group had a lower body mass index (BMI) than those in the cefdinir group, with no significant difference observed for other variables. Following 3 days of treatment, both the Pugongying and cefdinir groups showed a reduction in body temperature, VAS score, NE%, LYM%, and CRP level, with statistically significant differences within each individual group (*p* < 0.05). A statistically significant difference was noted in body temperature between the Pugongying group and cefdinir group, although the median body temperatures after treatment was 36.65 and 36.50, respectively, which had little clinical significance. No significant difference was found in the other clinical outcomes between the two groups after 3 days of treatment (*p* > 0.05), as shown in Appendix Table 1. Among the 127 breastmilk samples, 18 paired affected and healthy breastmilk samples were collected from 18 participants, and 36 pre- and post-treatment breastmilk samples were collected from 18 participants, including 9 women in the Pugongying group and 9 in the cefdinir group, for further PacBio sequencing at the species level.

**Table 1:** Participants’ demographic and other characteristics at baseline, as well as clinical outcomes.

Demographic characteristics	Total mean ± SD or M (IQR)	Pugongying group (*n* = 20) mean ± SD or M (IQR)	Cefdinir group (*n* = 17) mean ± SD or M (IQR)	*p* value
Age (years old)	31.64 ± 2.78	31.90 ± 2.34	30.94 ± 2.73	0.257
BMI (kg/m^2^)	22.10 (5.05)	20.63 (3.49)	23.03 (4.07)	0.006^*^
Educational level				> 0.999
Junior college and below	3	1	0	
Undergraduate	20	10	9	
Post-graduate student	19	9	8	
Participants’ characteristics				
Number of pregnancies				0.655
1	28	16	11	
2	9	2	3	
3	5	2	3	
Production times				> 0.999
1	36	18	15	
2	6	2	2	
Delivery mode				0.701
Spontaneous labor	30	15	14	
Caesarean birth	12	5	3	
Previous LM medical history				> 0.999
No	25	11	10	
Yes	17	9	7	
Chapped nipple				0.462
No	32	14	14	
Yes	10	6	3	
Clinical outcomes				
Temperature	38.20 (0.70)	38.30 (0.70)	38.00 (0.50)	0.198
VAS score	6.00 (2.00)	6.50 ± 1.57	5.76 ± 1.03	0.108
WBC	11.29 (5.06)	11.25 (5.04)	10.54 (4.19)	0.951
NE#	9.17 (4.74)	8.74 (5.74)	8.52 (4.31)	0.927
NE%	82.55 (9.95)	81.15 (10.75)	82.30 (9.70)	0.855
LYM#	1.23 (0.67)	1.39 ± 0.55	1.39 ± 0.34	0.972
LYM%	11.25 (5.50)	12.10 (6.90)	11.80 (6.05)	0.562
CRP	22.70 (32.15)	33.25 (26.35)	19.60 (37.05)	0.279

BMI, body mass index; CRP, C-reactive protein; IQR, inter quartile range; LM, lactational mastitis; LYM#, lymphocyte count; LYM%, percentage of lymphocytes; M, median; NE#, neutrophil count; NE%, percentage of neutrophils; SD, standard deviation; VAS, visual analogue scale; WBC, white blood cell count. * indicates that *p* < 0.05.

### 3.2. Microbial profiling by sequencing of 16S rRNA gene amplicons

The quantity of microbiota in the breastmilk samples collected via an aseptic protocol was sufficient for microbiota profiling based on 16S rRNA gene sequencing. All 127 samples yielded quantifiable PCR products and were sequenced. A total of 6,204,093 clean reads were generated, representing an average of 48,851 per sample. Reads were clustered at a similarity level of 97.0%.

### 3.3. Microbial communities of paired affected and healthy samples

In total, 42 paired affected and healthy samples were analyzed before the treatment. The breastmilk samples were analyzed to determine the composition and abundance of members of different phyla and genera. The rarefaction curve of the ACE index is used to verify whether the amount of sequencing data is sufficient to reflect the species diversity in the sample, and indirectly reflects the species richness in the sample. The curves of the genera from paired healthy and affected breastmilk samples approached the plateau phase, with more than 30,000 sequences per sample, indicating that the value was saturated, and there was no need to generate more sequences (Appendix Figure 1A).

**Figure 2:**
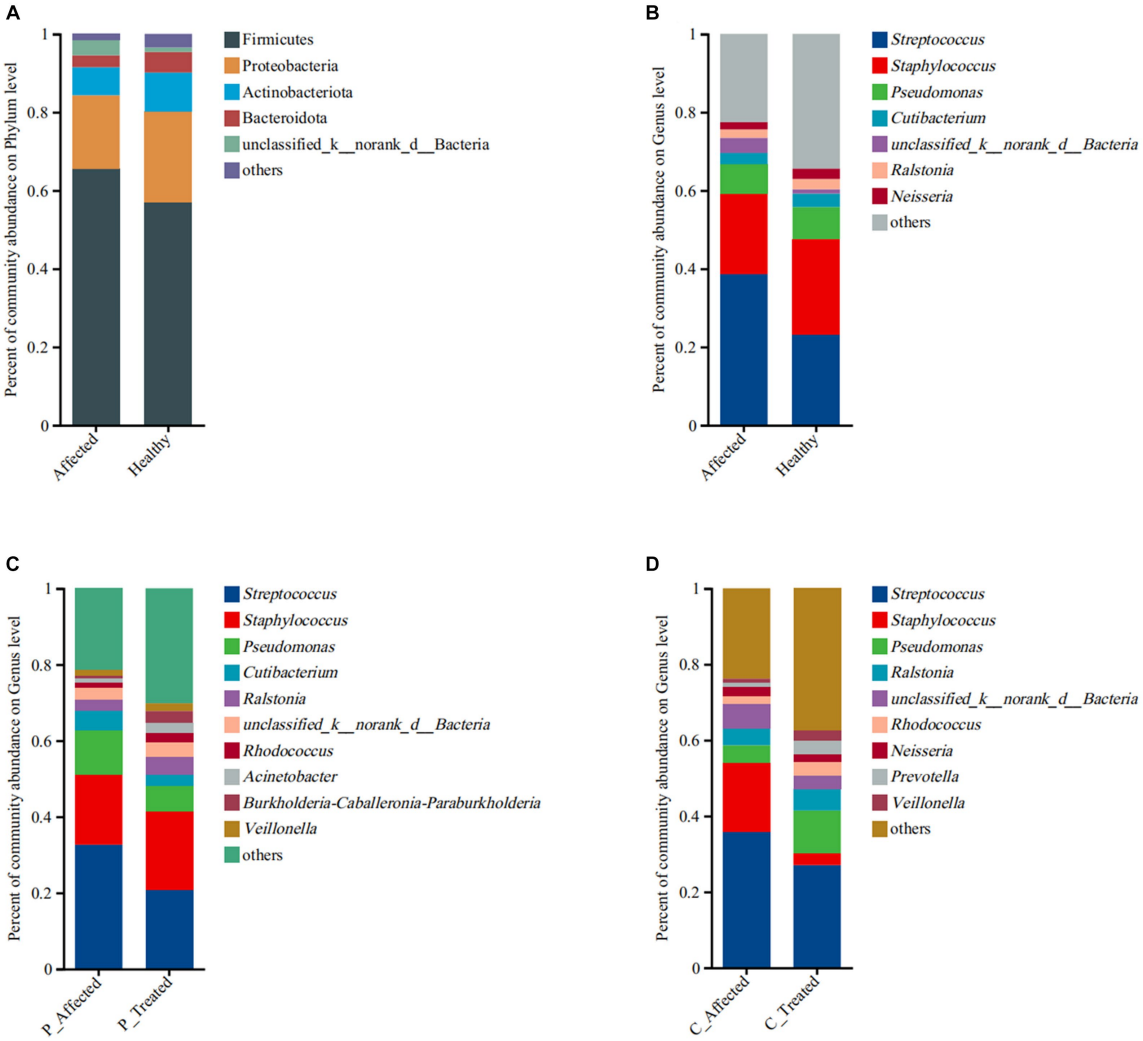
Comparison of microbial community abundance at the phylum level **(A)** and genus level **(B)** between paired breastmilk samples from healthy and affected breasts. Additionally, the figure presents a comparison of microbial community abundance at the genus level between pre- and post-treatment breastmilk samples from patients treated with Pugongying **(C)** and pre- and post-treatment breastmilk samples from patients treated with cefdinir **(D)**. P: Pugongying group; C: cefdinir group.

At the phylum level, the main microbes were Firmicutes, Proteobacteria, Actinobacterota, and Bacteroidota. The bacterial community composition and abundance are shown in Figure 2A. There were 1,667 genera in the breastmilk samples, with 62.93% (1,049/1,667) co-occurring. The main microbes were *Streptococcus*, *Staphylococcus*, and *Pseudomonas*. The bacterial community composition and abundance are presented in Figure 2B. There were more *Streptococcus* (38.58 vs. 23.22%) and fewer *Staphylococcus* (20.52 vs. 24.32%) in the affected breastmilk samples, while *Streptococcus* and *Staphylococcus* were present at virtually equivalent abundances in healthy breastmilk samples. At the species level, there were 653 species in the breastmilk samples, with 53.29% (348/653) of species co-occurring. *Streptococcus mitis* (14.71 vs. 2.77%) and *Streptococcus agalactiae* (9.94 vs. 6.93%) were more abundant, and *S. aureus* (18.14 vs. 23.17%) was less abundant in the affected breastmilk samples (Appendix Figure 2A).

**Figure 3:**
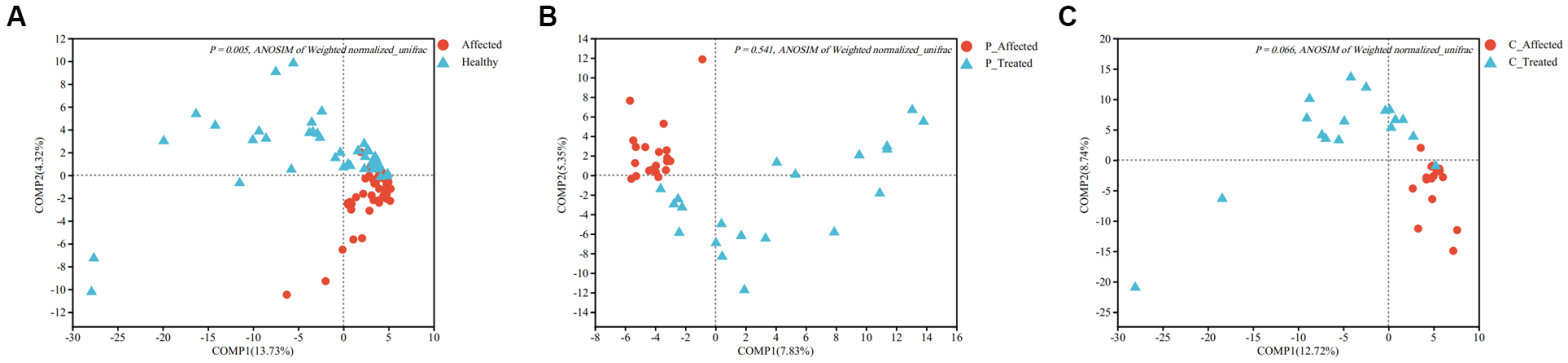
Partial least squares discriminant analysis (PLS-DA) comparing microbial communities between paired breastmilk samples from healthy and affected breasts **(A)**, between pre- and post-treatment breastmilk samples from patients treated with Pugongying **(B)** and between pre- and post-treatment breatmilk samples from patients treated with cefdinir **(C)**. P: Pugongying group; C: cefdinir group.

At the genus level, the PLS-DA results showed a clear visual distinction between clustering of the microbial communities of the paired healthy and affected samples. Furthermore, ANOSIM analysis based on Weighted normalized unifrac distance demonstrated a statistically significant difference in the structure of the healthy and affected breastmilk microbiomes (*p* = 0.005), as illustrated in Figure 3A. Notably, the first two components of Weighted normalized unifrac distance between the paired healthy and affected samples explained only a small percentage of the variability (13.73 and 4.32%, respectively). In terms of alpha diversity, both Shannon and Simpson indices revealed that breastmilk samples from the healthy breast had a higher microbial diversity than those from the affected breast. The differences between the paired samples were also statistically significant (Shannon: *p* = 0.006, Simpson: *p* = 0.005). Additional information is presented in Appendix Table 2.

**Figure 4:**
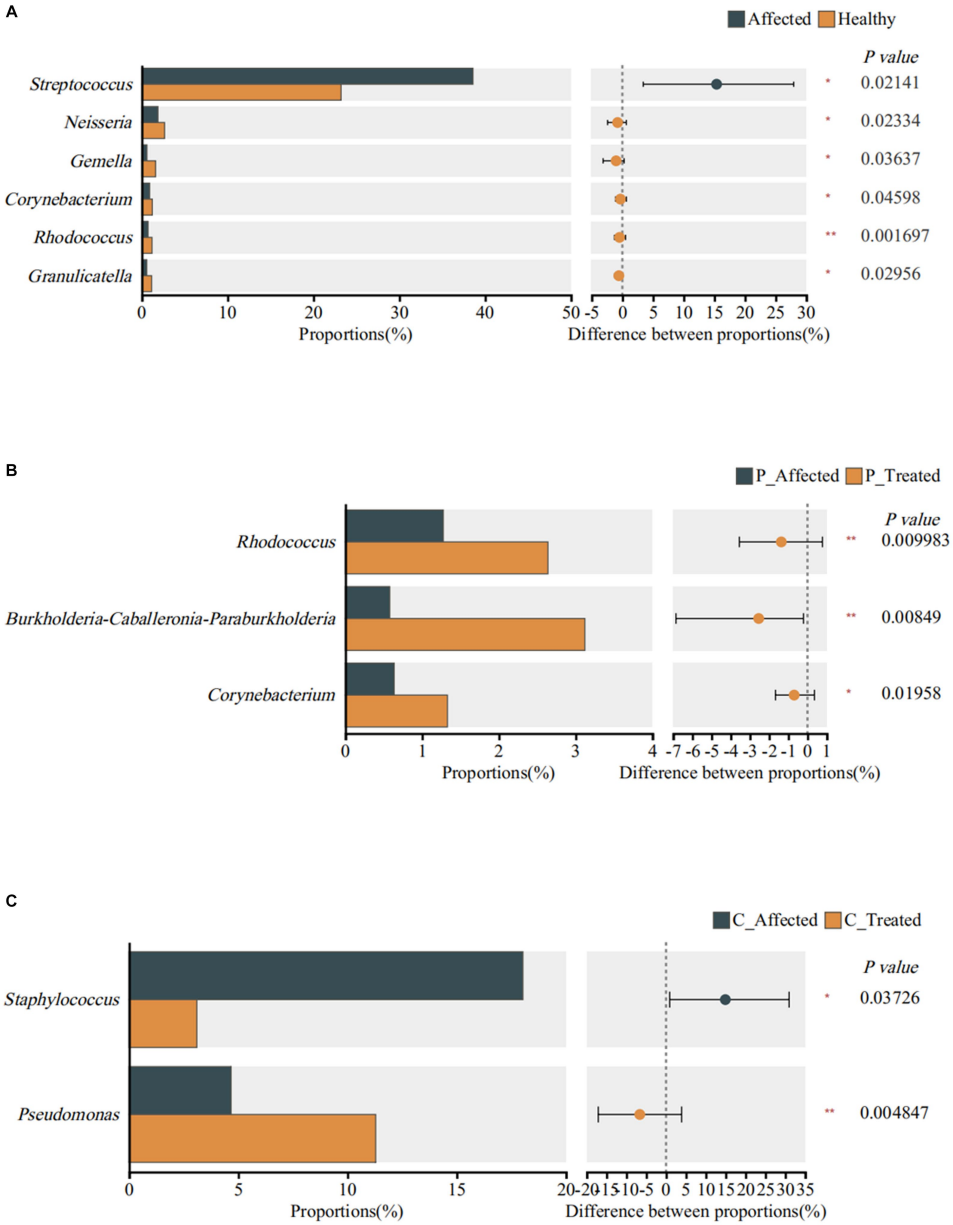
Identification of genera exhibiting significant differences between breastmilk samples from paired healthy and affected milk sample **(A)**, between pre- and post-treatment breastmilk samples from patients treated with Pugongyinge **(B)**, and between pre- and post-treatment breastmilk samples from patients treated with cefdinir capsule **(C)**. P: Pugongying group; C: cefdinir group.

At the genus level, the Wilcoxon signed-rank sum test revealed significant differences in the abundance of six genera. Specifically, *Streptococcus* exhibited a higher abundance in the affected breastmilk samples compared with healthy samples, with a statistically significant difference (*p* = 0.021). On the other hand, *Neisseria* (*p* = 0.023), *Gemella* (*p* = 0.036), *Corynebacterium* (*p* = 0.046), *Rhodococcus* (*p* = 0.002), and *Granmulicatella* (*p* = 0.03) exhibited higher abundance in healthy breastmilk samples (Figure 4A). At the species level, however, there was no species with significantly different abundance between paired affected and healthy breastmilk samples.

### 3.4. Microbial communities of the pre- and post-treatment samples from the Pugongying group

A total of 20 paired breastmilk samples (pre- and post-treatment) from the Pugongying group were analyzed. The microbial assessment was primarily conducted at the genus level. The rarefaction curve of the ACE index is used to verify whether the amount of sequencing data is sufficient to reflect the species diversity in the sample, and indirectly reflects the species richness in the sample. The curves of the genera from paired pre- and post-treatment breastmilk samples approached the plateau phase, with more than 28,000 sequences per sample, indicating that the value was saturated, and there was no need to generate more sequences (Appendix Figure 1B).

At the genus level, there were 1,390 genera in the breastmilk samples, with 58.13% (808/1,390) of genus co-occurring. In both pre- and post-treatment breastmilk samples from the Pugongying group, the main and commensal genera remained consistent, and their composition and abundance are depicted in Figure 2C. Compared with pre-treatment samples, post-treatment breastmilk samples exhibited a decrease in the abundance of *Streptococcus* (20.79 vs. 32.63%) and *Pseudomonas* (6.82 vs. 11.69%), while the abundance of *Staphylococcus* increased (20.55 vs. 18.47%). Notably, the distribution of *Streptococcus* and *Staphylococcus* in post-treatment samples was similar to that observed in healthy breastmilk samples, suggesting a balanced microbial community. At the species level, there were 484 species in the breastmilk samples, with 46.69% (226/484) of species co-occurring. *Streptococcus thermophilus* (46.21 vs. 26.92%) and *Pesudomonas marginalis* (2.89 vs. 0.19%) were more abundant, and *S. aureus* (18.09 vs. 30.84%) and *S. mitis* (2.95 vs. 10.21%) were less abundant, in the post-treatment breastmilk samples compared to the paired affected breastmilk samples. Notably, *S. agalactiae* was not observed in the post-treatment breastmilk samples (Appendix Figure 2B).

ANOSIM analysis of Weighted normalized unifrac distances showed that there was no statistically significant difference between the pre- and post-treatment samples (*p* = 0.541), despite the PLS-DA analysis showing clustering between pre- and post-treatment affected breastmilk samples in the Pugongying group, as illustrated in Figure 3B. Regarding the alpha diversity index, both Shannon and Simpson indices indicated that the microbial diversity of the post-treatment breastmilk samples was higher than that of the pre-treatment breastmilk samples, and this difference was statistically significant (Shannon: *p* = 0.018, Simpson: *p* = 0.012). Additional information is presented in Appendix Table 3.

At the genus level, the Wilcoxon signed rank-sum test showed that *Rhodococcus* (*p* = 0.01), *Burkholderia Caballeronia Paraburkholderia* (*p* = 0.008), and *Corynebacterium* (*p* = 0.02) exhibited significantly higher abundance in post-treatment samples (Figure 4B). At the species level, however, the results showed that *S. thermophilus* (*p* = 0.013) and *Streptococcus parasanguinis* (*p* = 0.022) exhibited higher abundance in the post-treatment breastmilk samples than in the pre-treatment samples.

Body temperature, VAS score, WBC, neutrophil count (NE#), NE%, lymphocyte count (LYM#), red blood cells (RBC), hemoglobin (HGB), platelets (PLT), and CRP level were retained, while LYM% was removed from a regression analysis of genera. Db-RDA analysis of Weighted normalized unifrac distance revealed a statistically significant correlation between VAS score and NE% and breastmilk composition (R^2^ = 0.22 and 0.22), as shown in Appendix Table 4 (*p* = 0.01 and 0.011, respectively). Additionally, the VAS score was found to have a negative correlation with *Corynebacterium* (*n* = 40, *p* < 0.001), while NE% was negatively correlated with both *Corynebacterium* (*n* = 40, *p* < 0.001) and *Collinsella* (*n* = 18, *p* = 0.037). Linear regression showed that there was no significant difference between the VAS score and Shannon index (R^2^ = 0.072, *p* = 0.052), VAS score and Simpson index (R^2^ = 0.071, *p* = 0.054), NE% and Shannon index (R^2^ = 0.037, *p* = 0.122), or NE% and Simpson index (R^2^ = 0.020, *p* = 0.187).

Body temperature, VAS score, WBC, NE#, LYM#, HGB, PLT, and CRP level were retained, while NE%, LYM%, and RBC were removed from a regression analysis of species. Db-RDA analysis of Weighted normalized unifrac distance showed that there was no significant correlation between clinical outcomes and species composition of the breastmilk microbial community (*p* > 0.05). As there was no significant correlation between alpha diversity metrics, clinical outcomes and microbial composition, linear regression and MaAsLin analyses were not conducted.

### 3.5. Microbial communities of pre- and post-treatment samples in the cefdinir group

The analysis involved 17 paired breastmilk samples from the cefdinir group, collected before and after treatment. The microbial assessment concentrated primarily on the genus level. The rarefaction curve of the ACE index is used to verify whether the amount of sequencing data is sufficient to reflect the species diversity in the sample, and indirectly reflects the species richness in the sample. The curves of the genus from paired pre- and post-treatment breastmilk samples approached the plateau phase, with more than 28,000 sequences per sample, indicating that the value was saturated, and there was no need to generate more sequences (Appendix Figure 1B).

At the genus level, there were 1,176 genera in the breastmilk samples, with 57.31% (674/1,176) of genus co-occurring. After cefdinir treatment, the overall composition of genera remained consistent with that of the pre-treatment samples, as displayed in Figure 2D. However, there were some notable differences in the abundance of specific genera. Specifically, the abundance of *Staphylococcus* decreased from 18.03% in pre-treatment samples to 3.1% in post-treatment breastmilk samples. On the other hand, the abundance of *Pseudomonas* increased in the post-treatment breastmilk samples (11.29 vs. 4.67%). At the species level, there were 510 species in the breastmilk samples, with 44.71% (228/510) of species co-occurring. *S. thermophilus* (19.63 vs. 10.31%), *P. marginalis* (5.62 vs. 0.91%) and *S. parasanguinis* (2.72 vs. 0.86%) were more abundant, and *S. aureus* (5.80 vs. 36.98%), *S. mitis* (9.31 vs.13.83%) and *S. agalaciae* (5.51 vs. 8.15%) were less abundant in the post-treatment breastmilk sample compared to paired affected breastmilk samples (Appendix Figure 2C).

ANOSIM of Weighted normalized unifrac distance showed no significant difference between paired samples (*p* = 0.066). However, PLS-DA revealed that there was clustering between pre- and post-treatment breastmilk samples in the cefdinir group (Figure 3C). Shannon and Simpson indices indicated that the microbial diversity of breastmilk samples after cefdinir treatment was higher compared with the breastmilk samples before cefdinir treatment, with a statistically significant difference (Shannon: *p* = 0.026, Simpson: *p* = 0.042). Additional information is presented in Appendix Table 5.

Wilcoxon signed rank-sum test showed that *Staphylococcus* was significantly less abundant in post-treatment samples (*p* = 0.037). *Pseudomonas* was significantly more abundant in post-treatment samples (*p* = 0.005), as shown in Figure 4C. However, there was no significant difference in species abundance between paired pre- and post-treatment breastmilk samples.

Body temperature, VAS score, WBC, NE%, LY#, RBC, HGB, PLT, and CRP level were retained, while LY% and NE# were removed from a regression analysis of genera. The DCA results indicated that the length of the axis was 3.16, which being less than the cutoff value of 4, precluded correlation analysis. Therefore, to perform correlation analysis, db-RDA of Weighted normalized unifrac distance was conducted. The results revealed that VAS score and WBC were correlated with breastmilk bacterial communities (R^2^ = 0.20 and 0.24, respectively), and the difference was statistically significant (*p* = 0.025 and 0.011, respectively), as shown in Appendix Table 6. VAS score was negatively correlated with *Microbacterium* (*n* = 30, *p* = 0.004) and *Tsukamurella* (*n* = 17, *p* = 0.039). WBC was negatively correlated with *Chryseobium* (*n* = 33, *p* = 0.016), *Paracoccus* (*n* = 25, *p* = 0.021), *Brevundimonas* (*n* = 34, *p* = 0.029), *Delftia* (*n* = 32, *p* = 0.039), and *Burkholderia Caballeronia Paraburkholderia* (*n* = 34, *p* = 0.048). Linear regression showed that no significant difference between VAS score and Shannon index (R^2^ = 0.019, *p* = 0.210), VAS score and Simpson index (R^2^ = 0.006, *p* = 0.279), WBC and Shannon index (R^2^ = 0.041, *p* = 0.130) and WBC and Simpson index (R^2^ = 0.060, *p* = 0.087).

Body temperature, VAS score, LYM#, RBC, HGB, PLT, and CRP level were retained, while WBC, NE%, NE#, and LYM% were removed from a regression analysis of species. Db-RDA analysis of Weighted normalized unifrac distance showed that there was no significant correlation between clinical outcomes and species composition of the breastmilk microbial community (*p* > 0.05). As there was no significant correlation between alpha diversity metrics, clinical outcomes and microbial composition, linear regression and MaAsLin analyses were not conducted.

## 4. Discussion

This study aimed to explore the etiology of LM by analyzing the microbial communities of paired affected and healthy breastmilk samples from women diagnosed with unilateral LM. Additionally, breastmilk samples were collected before and after treatment with Pugongying or cefdinir to assess the effects of Pugongying on clinical outcomes and breastmilk microbiome composition. The study was intended to provide a reference for a treatment regimen involving less antibiotic use and to establish a microbial basis for treating LM with Pugongying granules.

Although the microbial communities of breastmilk varied greatly among individuals in our study, the dominant species remained stable whether the breastmilk was collected from the healthy or affected breast or post-treatment. At the phylum level, the main phyla by abundance included Firmicutes, Proteobacteria, Actinobacterota, and Bacteroidota. At the genus level, *Streptococcus* and *Staphylococcus* were found to be the dominant microorganisms in breastmilk. Similar to previous studies which have suggested that breastmilk bacteria originate in the entero-mammary pathway or via the acquisition of “exogenously derived” bacteria (including maternal skin, infant oral cavity and pump-associated bacteria; Moossavi and Azad, 2019), this study found that the breastmilk microbiome had a composition similar to that of the intestinal microbiome and the external microbiome (including the skin and oral microbiome). A metagenomic analysis of microorganisms in the breastmilk and feces of healthy mothers showed that around 59.6% of the phyla present in lactating mothers’ feces were Firmicutes, which is similar to our findings that approximately 56.88% of the phyla detected in healthy breastmilk samples were Firmicutes (Ward et al., 2013). A previous study that assessed the correlation between the microbiomes of breastmilk and fecal samples from healthy lactating women by culture-independent methods showed that breastmilk and maternal feces had a strong canonical correlation (Williams et al., 2019). Additionally, we found gut-associated obligate anaerobes that have been recognized as potentially undergoing vertical mother-neonate transfer, such as *Bifidobacterium*, *Bacteroides*, *Dorea*, *Oscillibacter*, *Ruminococcus*, *Coprococcus*, *Faecalibacterium*, *Roseburia* and *Subdoligranulum* (data not shown; Jost et al., 2013b; Consales et al., 2022). Although we collected breastmilk samples under sterile conditions to eliminate the impact of skin and oral microorganisms, we still found unique nipple skin bacteria, such as *Lachnospira*, *Parabacteroides*, *Staphylococcus*, *Agathobacter* and *Lachnoclostridum* (Du et al., 2022), as well as skin bacteria, such as *S. mitis* (Biagi et al., 2018).

Previous studies based on culture-dependent approaches led to the belief that breastmilk was sterile (Gavin and Ostovar, 1977; McGuire and McGuire, 2017; Fernández and Rodríguez, 2020). However, subsequent research utilizing both culture-dependent and culture-independent methods has revealed that bacterial communities are in fact present in breastmilk. These studies have found significant differences in the composition of microbial communities in breastmilk, with *Staphylococcus* and *Streptococcus* being the most common microorganisms (Table 2).

**Table 2:** Characteristics of microbial communities in milk samples from healthy mothers.

Major species	Sequencing methods	Sample size	Country
*Staphylococcus/Streptococcus* (Hunt et al., 2012)	Pyrophosphate sequencing	16	USA
*Staphylococcus/Streptococcus/Pseudomonas/Ralstonia* (Jost et al., 2013a)	Pyrophosphate sequencing	7	Switzerland
*Pseudomonas/Staphylococcus* (Ward et al., 2013)	Metagenomics	10	Canada
*Staphylococcus/Streptococcus* (Jiménez et al., 2015)	Illumina sequencing	10	Spain
*Staphylococcaceae/Streptococcaceae/Pseudomonadaceae/Enterobacteriaceae* (Cabrera-Rubio et al., 2016)	Pyrophosphate sequencing	10	Spain
*Staphylococcus/ Lactobacillus /Pseudomonas/Streptococcus* (Urbaniak et al., 2016)	Illumina sequencing	39	USA
*Staphylococcus/Streptococcus* (Sakwinska et al., 2016)	Illumina sequencing	34	China

The present study demonstrated that *Pseudomonas* was highly abundant in the breastmilk samples, dominating healthy, affected, and post-treatment samples. While previous studies have suggested that the presence of *Pseudomonas* could be due to DNA extraction kits or laboratory-related contamination, or the use of non-sterile breast pumps, our findings suggest that *Pseudomonas* abundance changes as LM progresses and is treated (Salter et al., 2014; Jiménez et al., 2017). Although we did not include a blank control or attempt to remove contaminating bacteria, it is unlikely that environmental contamination alone would explain the observed changes in *Pseudomonas* abundance. Notably, *Staphylococcus/S. aureus* and *Pseudomonas/ P. marginalis* abundance changed in opposite directions in all comparisons. Previous research has shown that *Pseudomonas. aeruginosa* regulates the synthesis of multiple extracellular products, inhibiting *S. aureus* growth through quorum-sensing in diseases, such as chronic obstructive pulmonary disease and cystic fibrosis (Li et al., 2021). However, the interactions between *Pseudomonas* and *Staphylococcus*, as well as the role of *Pseudomonas* in breastmilk from patients with LM, require further investigation.

This study found that samples from the affected breast exhibited lower microbial diversity and higher *Streptococcus* abundance compared with samples from the healthy breast. Previous studies exploring the etiology of LM relied on culture-dependent approaches, which suggested *Staphylococcus* as the primary pathogenic bacterium (El-Mohandes et al., 1993). However, subsequent research revealed that breastmilk microbial communities are composed of several symbiotic bacteria, leading to the suggestion that LM could be caused by opportunistic pathogens (Jiménez et al., 2015). Two cross-sectional studies, one based on culture-dependent methods and another on culture-independent methods, support the association of *Streptococcus* with LM (Delgado et al., 2008; Mediano et al., 2017).

Post-treatment breastmilk samples from both the Pugongying and the cefdinir groups exhibited a decrease in *Streptococcus* abundance. However, these differences were not significant, which could be related to high heterogeneity among *Streptococcus* strains. It is essential to identify specific bacterial traits for proper species identification, as *Streptococcus* comprises both symbiotic and pathogenic strains. There are limitations to the ability of phenotype-based methods to identify these traits, and overlapping sequences among *Streptococcus* strains make it challenging to detect them exclusively by short-length 16S rRNA gene sequencing (Ikryannikova et al., 2011; Teles et al., 2011; Martin et al., 2016). Thus, we performed full-length 16S rRNA gene sequencing to further investigate potential pathogens at the species level. We found that the abundance of *S. mitis* and *S. agalactiae* may be related to LM occurrence. Additionally, *S. thermophilus* and *S. parasanguinis* could play important role in recovery from LM. Alternative methods, such as sequencing housekeeping genes and using matrix-assisted laser desorption ionization time-of-flight mass spectrometry (MALDI-TOF VITEK MS), also aim to overcome this challenge and improve the accuracy of species identification. One study used MALDI-TOF VITEK MS to analyze the antimicrobial susceptibility profile of the culturable microbiome isolated from breastmilk samples collected from patients with LM. Coagulase-negative *Staphylococcus*, mainly *S. aureus*, and *Streptococcus*, including *Streptococcus mitis/oralis*, *Streptococcus salivarius*, and *S. parasanguinis*, were identified as the main species in LM breastmilk samples (Marin et al., 2017). However, the etiology of these species was not identified. The potential role of *Streptococcus* in LM pathogenesis has attracted clinicians’ attention due to its clinical manifestations and lack of suitable treatments (Contreras and Rodriguez, 2011; Tena et al., 2016; Hald and Schonheyder, 2018). Due to the small sample size included in our study, accurate identification of *Streptococcus* strains and corresponding treatment regimens should be explored further in larger studies in the future.

Changes in *Corynebacterium* abundance may also be associated with LM occurrence. In this study we found that compared with breastmilk samples from the healthy breast, samples from the affected breast exhibited lower *Corynebacterium* abundance. In terms of the correlation between clinical outcomes and bacteria, *Corynebacterium* was present in all breastmilk samples from women in the Pugongying group, and a negative correlation was found between *Corynebacterium*, VAS score, and NE%, suggesting that *Corynebacterium* may protect against LM. Culture-independent studies have previously identified *Corynebacterium* as a potential LM pathogen, and the cooperative relationship between *Staphylococcus* and *Corynebacterium* may lead to LM, as its growth is not inhibited by other bacteria in breastmilk (Heikkila and Saris, 2003; Mediano et al., 2017). Additionally, *Corynebacterium* has been associated with granulomatous mastitis (Dobinson et al., 2015; Fernández-Natal et al., 2016). Similar to *Streptococcus*, accurate identification of *Corynebacterium* strains remains challenging; however, the PacBio sequencing results did not include *Corynebacterium* strains (Barberis et al., 2014). Discrepancies in the results between our and previous studies may have arisen due to the different sample sources. Furthermore, previous studies either did not include a control group of samples from the LM-affected breast or used healthy mothers as the control group. Thus, the role of *Corynebacterium* in LM may vary depending on the species. One study reported that dietary factors, such as saturated fatty acids and monounsaturated fatty acids, can affect *Corynebacterium* abundance (Fernandez et al., 2020). Future research under controlled dietary conditions is needed to accurately identify *Corynebacterium* species and investigate their role in LM pathogenesis and treatment.

The results from this study indicated that treatment with cefdinir or Pugongying increased the microbial diversity of breastmilk. However, it should be noted that different microorganisms were affected by the two different treatments, particularly *Staphylococcus*. Dandelion (Pugongying) has been found to inhibit the growth of *S. aureus in vitro*, which is consistent with our analysis of post-cefdinir treatment samples (Chen et al., 2021). However, it is important to consider that culture-dependent methods may be more conducive to the growth of *Staphylococcus*, leading to an underestimation of the impact of other bacteria (Ding et al., 2019; Fernandez et al., 2020). On the other hand, 16S rRNA methods for accurate quantification of live bacterial cells may overestimate the presence of low-abundance microorganisms, such as *Rhodococcus* and *Burkholderia Caballeronia Paraburkholderia*, due to the presence of dead bacteria, human DNA, or environmental contaminants (Louca et al., 2018). To address this issue, mock microbial dilution series may be useful.

To our knowledge, no previous studies have explored the effect of Chinese herbs on breastmilk microbial communities in women diagnosed with LM. The aim of this study was to analyze the microbial communities in pre- and post-treatment breastmilk samples in patients treated with Pugongying to provide a basis for using Pugongying to treat LM from a microbiological perspective.

There were some limitations to the present study. First, as breastmilk was not collected from healthy lactating women, it is possible that some of the changes we observed in the microbial communities occurred prior to the onset of LM symptoms. However, the clear differences in microbial community composition between paired breastmilk samples from healthy and affected breasts suggests that the alterations that we observed were indeed due to LM. Another consideration is that we did not include blank controls in our sequencing analyses, raising the possibility that contaminants could have impacted the amplification of low-abundance microorganisms; however, no common environmental contaminants were identified in our sequencing analyses, suggesting that this was not a factor. In addition, dietary intake, which can influence the abundance of specific microorganisms, such as *Gemella* and *Corynebacterium* was not controlled in this study, although this would have been largely infeasible in this study population.

## 5. Conclusion

In conclusion, LM could be associated with a reduction in microbial diversity and an increase in the abundance of *Streptococcus* in breastmilk. We showed that Pugongying enhances the microbial diversity of breastmilk. However, given the variability and diversity of individual microorganisms, further research is required to explore the role of *Streptococcus* and *Corynebacterium* in LM pathogenesis and alternative treatment. Prospective long-term follow-up cohort studies are essential to provide additional insight into the species that contribute to LM onset and the impact of Pugongying on the breastmilk microbial community.

## Data availability statement

The datasets presented in this study can be found in online repositories. The names of the repository/repositories and accession number(s) can be found at: NCBI - PRJNA999935 and NVBI - PRJNA1027787.

## Ethics statement

The studies involving humans were approved by Ethics Committee of Beijing University of Chinese Medicine Third Affiliated Hospital (Approval No. BZYSY-SFKTPJ-1). The studies were conducted in accordance with the local legislation and institutional requirements. The participants provided their written informed consent to participate in this study.

## Author contributions

XP and XJ contributed to the conception of the work. JL and XJ contributed to the design of the work. JX, XJ, YF, YX, and NC contribute to acquisition of data for the work. XJ contributes to analysis and interpretation of data for the work. XJ and JX drafted the manuscript. CL, WM, and XX revised the manuscript. All authors contributed to the article and approved the submitted version.

## Footnotes

^1^ https://cloud.majorbio.com

^2^ https://cran.r-project.org/web/packages/vegan/index.html

## Glossary

ANOSIM: Analysis of similarities
BMI: Body mass index
CFDA: Chinese Food and Drug Administration
CRP: C-reactive protein
Db-RDA: distance-based redundancy analysis
HGB: Hemoglobin
IQR: Inter quartile range
LM: Lactational mastitis
LYM%: Percentage of lymphocytes
LYM#: Lymphocyte count
MALDI-TOF VITEK MS: Matrix-assisted laser desorption ionization time-of-flight mass spectrometry
MaAsLin: Multivariate Association with Linear Models
M: Median
NE%: Percentage of neutrophils
NE#: Neutrophil count
OTU: Operational taxonomic unit
PLS-DA: Partial least squares discriminant analysis
PLT: Platelets
RBC: Red blood cell
SD: Standard deviation
Sobs: Observed species
VAS: Visual analogue scale
VIF: Variance inflation factor
WBC: White blood cell count
