# Supplementary material for: Breastmilk microbiome changes associated with lactational mastitis and treatment with dandelion extract
Source: Front Microbiol. 2023 Nov 13;14:1247868. doi: 10.3389/fmicb.2023.1247868 (PMC10679338; doi:10.3389/fmicb.2023.1247868)
Supplement: Supplementary file 1 [file Data_Sheet_1.docx]

Supplementary Material

Breastmilk microbiome changes associated with lactational mastitis and treatment with dandelion extract

Xinyan Jin†, Jinhe Xiao†, Chunli Lu, Wenxin Ma, Yingyi Fan, Xue Xue, Yaru Xia, Nana Chen

*** Correspondence:** Corresponding Author: Jianping Liu liujp@bucm.edu.cn, Xiaohua Pei pxh_127@163.com

# Supplementary Figures and Tables

**Supplementary figures:**

**
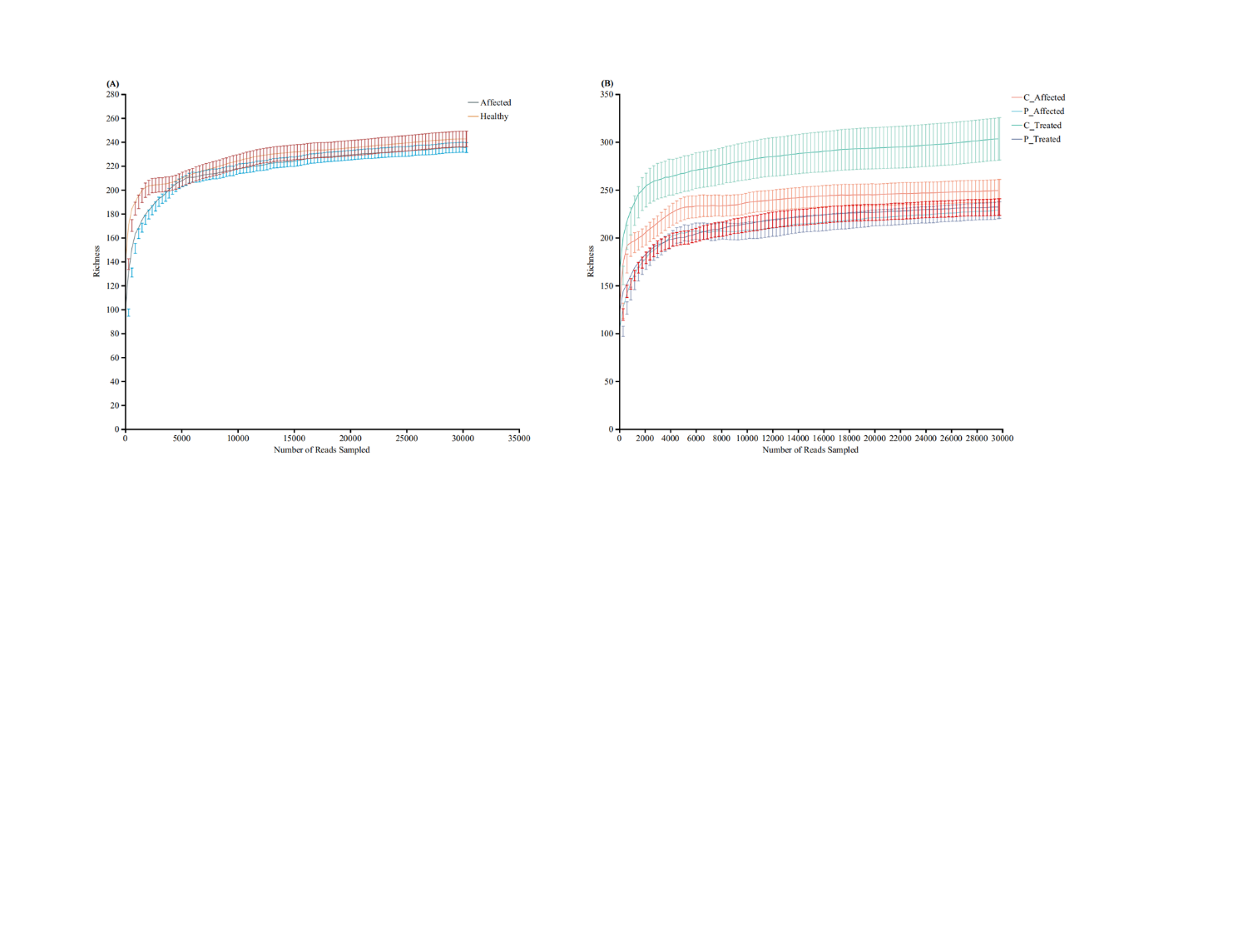
**

Appendix Figure 1. The rarefaction curve of the ACE index of paired healthy and affected breastmilk samples (A), of pre- and post-treatment with either Pugongying or cefdinir (B). P: Pugongying group; C: cefdinir group.


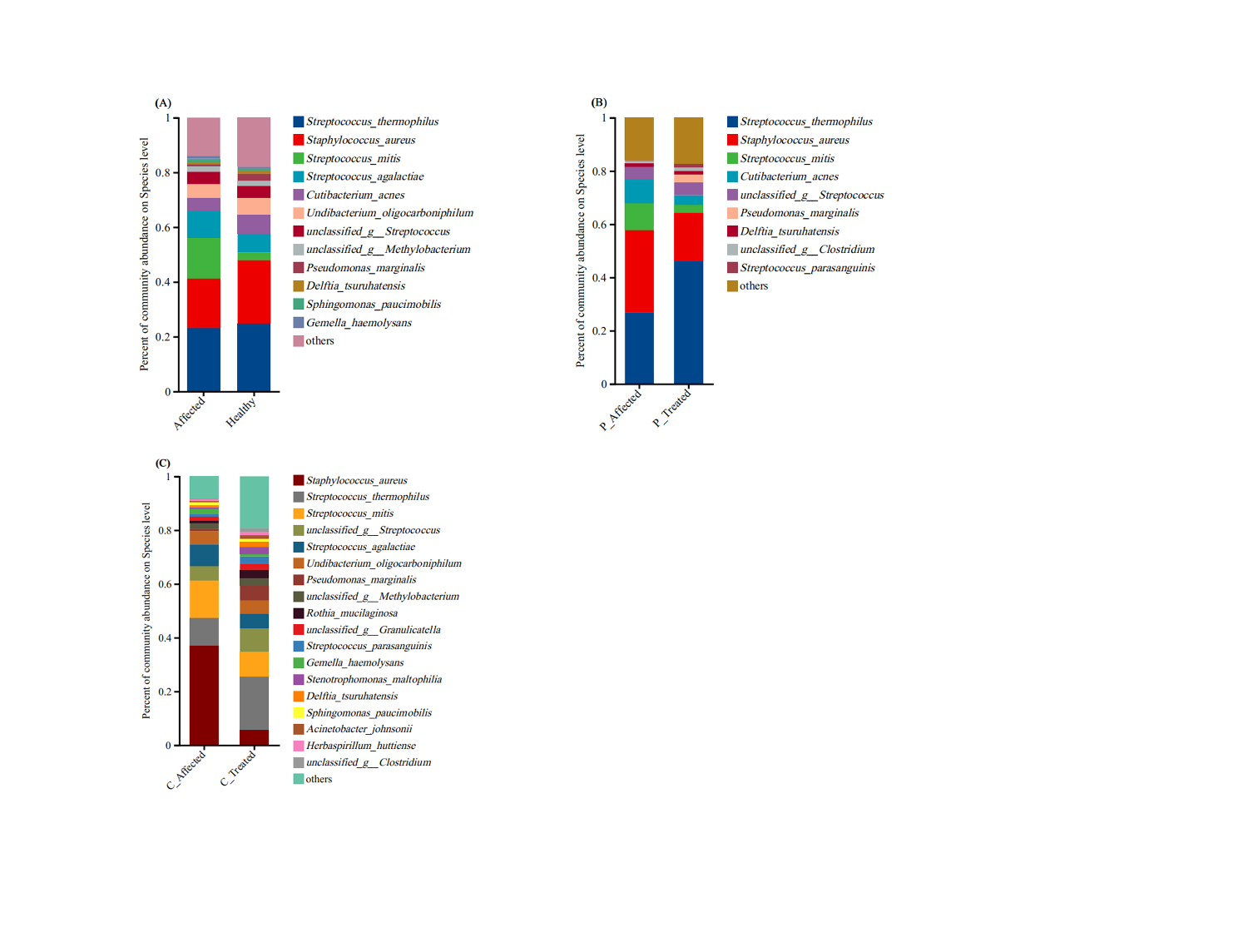


Appendix Figure 2. Comparison of microbial community abundance at the species level between paired breastmilk samples from healthy and affected breasts (A), between pre- and post-treatment breastmilk samples from patients treated with Pugongying (B) and pre- and post-treatment breastmilk samples from patients treated with cefdinir (C). P: Pugongying group; C: cefdinir group.

**Supplementary tables:**

Table 1. Comparison of clinical outcomes after 3-day treatment between the Pugongying group and the cefdinir group

Table 2. Comparison of alpha diversity between the paired affected and healthy breastmilk samples

Table 3. Comparison of alpha diversity between paired pre- and post-treatment breastmilk samples in the Pugongying group

Table 4. Db-RDA of bacterial composition and clinical outcomes of pre- and post-treatment breastmilk samples in the Pugongying group

Table 5. Comparison of alpha diversity between paired pre- and post-treatment breastmilk samples in the cefdinir group

Table 6. Db-RDA of bacterial composition and clinical outcomes of pre- and post-treatment breastmilk samples in the cefdinir group

Table 1. Comparison of clinical outcomes after 3-day treatment between the Pugongying group and the cefdinir group

|  | Group P (n = 20) | | *P_1_* | Group C (n = 17) | | *P_2_* | *P_3_* |
| --- | --- | --- | --- | --- | --- | --- | --- |
|  | Pre- treatment  Mean ± SD or M (IQR) | Post- treatment  Mean ± SD or M (IQR) |  | Pre- treatment  Mean ± SD or M (IQR) | Post- treatment  Mean ± SD or M (IQR) |  |  |
| Temperature | 38.30 (0.70) | 36.65 (0.40) | <0.001 | 38.00 (0.50) | 36.50 (0.80) | <0.001 | 0.03* |
| VAS score | 6.50 (3.00) | 3.00 (2.00) | <0.001 | 6.00 (2.00) | 4.00 (3.00) | <0.001 | 0.127 |
| WBC | 11.25 (5.04) | 6.00 (1.68) | <0.001 | 10.54 (4.19) | 5.66 (1.45) | <0.001 | 0.721 |
| NE# | 8.74 (5.74) | 3.45(1.63) | 0.001 | 8.52 (4.31) | 3.43 (1.14) | <0.001 | 0.796 |
| NE% | 81.15 (10.75) | 55.90 (8.12) | 0.001 | 82.30 (9.70) | 55.50 (9.00) | 0.005 | 0.772 |
| LYM# | 1.39 ± 0.55 | 1.78 ± 0.42 | < 0.001 | 1.39 ± 0.34 | 1.86 ± 0.43 | <0.001 | 0.578 |
| LYM% | 12.10 (6.90) | 32.04 (8.43) | <0.001 | 11.80 (6.05) | 32.40 (9.35) | <0.001 | 0.729 |
| CRP | 33.25 (26.35) | 16.30 (13.12) | 0.014 | 19.60 (37.05) | 12.90 (13.55) | 0.042 | 0.266 |

Notes: P_1_: Comparison of the Pugongying group before and after treatment; P_2_: Comparison of the cefdinir group before and after treatment; P_3_: Comparison between the Pugongying and cefdinir groups after 3-day treatment. Group P: Pugongying group; Group C: cefdinir group. CRP: C-reactive protein; IQR: inter quartile range; LYM#: lymphocyte count; LYM%: percentage of lymphocytes; M: median; NE#: neutrophil count; NE%: percentage of neutrophils; SD: standard deviation; VAS: visual analogue scale; WBC: white blood cell count

Table 2. Comparison of alpha diversity between the paired affected and healthy breastmilk samples

|  | Healthy, M (SD) | Affected, M (SD) | *P* value |
| --- | --- | --- | --- |
| Sobs index | 303.71 (215.8) | 264.45 (133.92) | 0.464 |
| Shannon index | 2.474 (1.231) | 1.746 (1.042) | 0.006 |
| Simpson index | 0.313 (0.243) | 0.471 (0.279) | 0.005 |
| ACE index | 345.66 (226.96) | 334.59 (146.44) | 0.644 |
| Chao index | 339.51 (231.56) | 323.63 (146.92) | 0.745 |
| Coverage index | 0.999 (0.001) | 0.998 (0.001) | < 0.001 |

Notes: M: mean; SD: standard deviation

Table 3. Comparison of alpha diversity between paired pre- and post-treatment breastmilk samples in the Pugongying group

|  | Pre-treatment, M (SD) | Post-treatment, M (SD) | *P* value |
| --- | --- | --- | --- |
| Sobs index | 188.4 (84.378) | 202 (70.544) | 0.370 |
| Shannon index | 1.728 (0.876) | 2.354 (0.733) | 0.018 |
| Simpson index | 0.436 (0.275) | 0.265 (0.193) | 0.012 |
| ACE index | 228.86 (90.036) | 232.02 (88.086) | 0.780 |
| Chao index | 222.35 (96.928) | 228.12 (81.966) | 0.808 |
| Coverage index | 0.999 (0.001) | 0.999 (0.001) | 0.025 |

Notes: M: mean; SD: standard deviation

Table 4. Db-RDA of bacterial composition and clinical outcomes of pre- and post-treatment breastmilk samples in the Pugongying group

|  | CAP1 | CAP2 | R^2^ | *P* value |
| --- | --- | --- | --- | --- |
| Temperature | -0.9619 | 0.2735 | 0.0804 | 0.216 |
| VAS score | -0.9973 | -0.0735 | 0.2248 | 0.01 |
| WBC | -0.9827 | 0.185 | 0.0989 | 0.151 |
| NE# | 0.9997 | -0.0255 | 0.0655 | 0.29 |
| NE% | -0.7833 | 0.6217 | 0.2189 | 0.011 |
| LYM# | 0.0304 | -0.9995 | 0.091 | 0.159 |
| RBC | 0.1651 | 0.9863 | 0.0268 | 0.598 |
| HGB | -0.1288 | -0.9917 | 0.1594 | 0.025 |
| PLT | 0.1448 | -0.9895 | 0.0261 | 0.615 |
| CRP | -0.9984 | -0.0558 | 0.1494 | 0.05 |

Notes: CRP: C-reactive protein; HGB: hemoglobin; LYM#: lymphocyte count; NE%: percentage of neutrophils; NE #: neutrophil count; PLT: platelets; RBC: red blood cell; VAS: visual analogue scale; WBC: white blood cell count

Table 5. Comparison of alpha diversity between paired pre- and post-treatment breastmilk samples in the cefdinir group

|  | Pre-treatment, M (SD) | Post-treatment, M (SD) | *P* value |
| --- | --- | --- | --- |
| Sobs index | 220.53 (92.486) | 275.88 (174.16) | 0.102 |
| Shannon index | 2.077 (0.957) | 2.805 (1.034) | 0.026 |
| Simpson index | 0.352 (0.248) | 0.211 (0.198) | 0.042 |
| ACE index | 249.22 (101.01) | 303.29 (191.64) | 0.368 |
| Chao index | 247.15 (102.15) | 301.22 (195.79) | 0.368 |
| Coverage | 0.999 (0.001) | 0.999 (0.001) | 0.245 |

Notes: M: mean; SD: standard deviation

Table 6. Db-RDA of bacterial composition and clinical outcomes of pre- and post-treatment breastmilk samples in the cefdinir group

|  | CAP1 | CAP2 | R^2^ | *P* value |
| --- | --- | --- | --- | --- |
| Temperature | -0.1732 | 0.9849 | 0.1254 | 0.126 |
| VAS score | 0.2454 | 0.9694 | 0.2013 | 0.025 |
| WBC | -0.1483 | 0.9889 | 0.2434 | 0.011 |
| NE% | 0.0878 | 0.9961 | 0.1266 | 0.141 |
| LYM# | 0.708 | 0.7062 | 0.0738 | 0.294 |
| RBC | -0.4181 | -0.9084 | 0.3812 | 0.021 |
| HGB | 0.4918 | 0.8707 | 0.0981 | 0.215 |
| PLT | 0.9723 | 0.2339 | 0.1477 | 0.092 |
| CRP | -0.0294 | 0.9996 | 0.0175 | 0.774 |

Notes: CRP: C-reactive protein; HGB: hemoglobin; LYM#: lymphocyte count; NE%: percentage of neutrophils; PLT: platelets; RBC: red blood cell; VAS: visual analogue scale; WBC: white blood cell count
